# A novel monocyte-based biomarker of cardiovascular risk: comparison with traditional cardiovascular risk calculators

**DOI:** 10.1016/j.ajpc.2026.101663

**Published:** 2026-05-05

**Authors:** Fatemah Almarri, Shuaihua Qiao, Jiale Gao, Li'na Kang, Soundrie Padayachee, Biao Xu, Ashish Patel, Albert Ferro

**Affiliations:** a School of Cardiovascular and Metabolic Medicine and Sciences, British Heart Foundation Centre of Research Excellence, King’s College London (Waterloo Campus), 3.07 Franklin-Wilkins Building, 150 Stamford Street, London SE1 9NH, UK; b Department of Cardiology, Nanjing Drum Tower Hospital, The Affiliated Hospital of Nanjing University Medical School, Nanjing University, Nanjing, China; c Department of Ultrasonic Angiology, Guy’s & St Thomas’ Hospitals, London, UK

## 1 Introduction

Atherosclerotic cardiovascular disease remains the leading global cause of mortality despite advances in prevention. A major challenge is identifying individuals with subclinical, asymptomatic atherosclerosis, which is highly prevalent even in apparently healthy people [1]. Although imaging modalities such as computed tomography angiography and carotid ultrasonography can detect early disease, they are not feasible for large-scale screening.

Current cardiovascular risk calculators, including QRISK3 and related models, estimate risk using clinical and demographic variables [2]. While effective at a population level, they perform less reliably at the individual level and may misclassify risk, particularly in older populations [3,4]. These limitations arise because such models do not capture the biological processes underlying early atherogenesis, highlighting the need for biomarkers that directly reflect disease activity.

Inflammation is central to atherosclerosis, with monocytes playing a key role in plaque development [5]. Circulating CD16-expressing monocyte subsets and monocyte–platelet aggregates (MPAs) are elevated in symptomatic disease and associated with adverse outcomes [6]. However, their utility in detecting early, asymptomatic disease has not been established.

This study aimed to determine whether circulating monocyte phenotype and MPAs can identify subclinical atherosclerosis and outperform conventional cardiovascular risk calculators in this regard.

## 2 Methods

### 2.1 Study populations

#### 2.1.1 Discovery cohort

The study was approved by HRA and Health and Care Research Wales (research ethics committee reference 21/NE/0189). Asymptomatic adults (*n* = 39) were recruited either by advertisement or sequentially from patients attending the Hypertension Clinics at Guy’s and St Thomas’ Hospitals, London, UK. Exclusion criteria were: age<18 years, history of cardiovascular disease (other than hypertension), ingestion of any medication within the preceding 2 weeks, pregnancy, or inability/unwillingness to provide informed consent. All participants underwent a clinical history, physical examination and electrocardiography to exclude overt cardiovascular or other systemic disease. Blood pressure was measured in the sitting position, after a resting period of 5 min, using a validated oscillometric device (Omron IntelliIT, Omron Corporation, Japan). Three readings were obtained at two-minute intervals, and the mean of the second and third values was used for analysis. Venous blood (50 mL) was collected for full blood count and routine biochemistry, these being undertaken by Synnovis Laboratories (St Thomas’ Hospital, London, UK), and for flow cytometric analysis. Carotid ultrasonography was performed to assess carotid intima–media thickness (cIMT) and the presence or absence of plaque. Ten-year cardiovascular risk was estimated using QRISK3 (https://qrisk.org).

#### 2.1.2 Validation cohort

The study was approved by the Medical Ethics Committee of Nanjing Drum Tower Hospital, Medical School of Nanjing University, Nanjing, China (ethics committee reference 2025-0115-02). Healthy adults (*n* = 151) were recruited from the Physical Examination Centre at Drum Tower Hospital, where routine medical evaluations are performed for employment or health screening. Exclusion criteria and screening procedures were identical to those used in the discovery cohort. Blood sampling and carotid ultrasonography were performed using the same protocols, with the exception that haematological and biochemical analyses were conducted in the hospital’s pathology laboratories. Ten-year cardiovascular risk was calculated using China-PAR (https://doi.org/10.3760/cma.j.issn.0253-9624.2019.01.004).

Table 1 shows the demographic characteristics of recruited subjects.

**Table 1:** Subject demographics according to source of recruitment.

	Discovery cohort: hypertension clinic	Discovery cohort: advertisement	Validation cohort
Number (male / female)	14/12	5/8	76/75
Age (years)	41.1 ± 16.3	39.6 ± 11.7	47.0 ± 10.5
Smokers	14	6	35
Systolic blood pressure (mmHg)	141.6 ± 10.5	123.9 ± 7.1	123.5 ± 15.4
Diastolic blood pressure (mmHg)	88.7 ± 10.3	78.8 ± 2.1	78.5 ± 10.3
Creatinine (μmol/L)	76.4 ± 15.9	75.7 ± 19.2	67.9 ± 15.7
HbA_1c_ (%)	5.77±0.10	5.63±0.25	5.56±0.75
Total cholesterol (mmol/L)	4.95±1.06	5.38±0.73	4.92±0.92
LDL-cholesterol (mmol/L)	3.04±0.93	3.47±0.72	3.10±0.81
HDL-cholesterol (mmol/L)	1.39±0.42	1.44±0.23	1.41±0.38
Triglycerides (mmol/L)	1.42±0.96	1.07±0.34	1.60±0.64
Haemoglobin (g/L)	140.0 ± 12.8	129.4 ± 16.0	141.2 ± 17.0
Total white cell count (× 10^9^/L)	6.14±1.41	5.44±1.69	5.96±1.51
Neutrophil count (× 10^9^/L)	3.73±1.20	3.19±1.26	3.21±1.22
Monocyte count (× 10^9^/L)	0.48±0.15	0.45±0.14	0.34±0.12
Lymphocyte count (× 10^9^/L)	1.67±0.47	2.07±0.61	1.94±0.52
Platelet count (× 10^9^/L)	254.0 ± 13.8	240.1 ± 54.0	238.2 ± 64.2
Medications			
Beta blockers	3		
Alpha blockers	2		
Calcium channel blockers	8		
Renin-angiotensin system blockers	14		
Aspirin	2		
Diuretics	6		
Statins	8		

#### 2.1.3 Flow cytometry

Peripheral blood was processed within 15–30 min of venesection. Whole blood (100 µl) was incubated with 20 µl Human BD Fc-block (BD Pharmingen™) for 10 min on ice, and then stained in the dark at 4 °C for 30 min with 5 µl of the following fluorochrome-conjugated antibodies: PE-labelled anti- CD14, FITC-labelled anti- CD16, and APC-labelled anti-CD42b (all BD Pharmingen). Erythrocytes were then lysed using Pharm Lyse (BD Biosciences) and washed using flow cytometry buffer (PBS, 0.5 % [w/v] BSA, 2 mM EDTA). Stained cells were fixed in 4 % paraformaldehyde and analysed within 72 h on a MACSQuant Flow Cytometer (Miltenyi Biotec). Fluorescence-minus-one (FMO) controls were used to define positive gates for each marker. Data analysis was performed using FlowJo v10 (FlowJo LLC) by a researcher blinded to clinical information. Monocytes were classified into classical (CD14^++^CD16^−^), intermediate (CD14^++^CD16^+^), and non-classical (CD14^low^CD16^+^) subsets. MPAs were defined as CD14^+^CD42b^+^ events.

#### 2.1.4 Composite biomarker

The Monocyte Atherosclerotic Risk Score (MARS) was derived as the ratio of MPAs to classical monocytes.

#### 2.1.5 Carotid ultrasonography

cIMT and plaque presence were assessed using standardised duplex ultrasonography. Participants were classified as having high-risk cIMT if values were at or above the 75th percentile for age and sex. Plaque was defined as a focal lesion >1.5 mm.

### 2.2 Statistical analysis

Associations were evaluated using linear regression. Predictive performance was assessed using receiver operating characteristic (ROC) analysis and area under the curve (AUC). Statistical significance was defined as *P* < 0.05.

## 3 Results

### 3.1 Discovery cohort

**Fig. 1:**
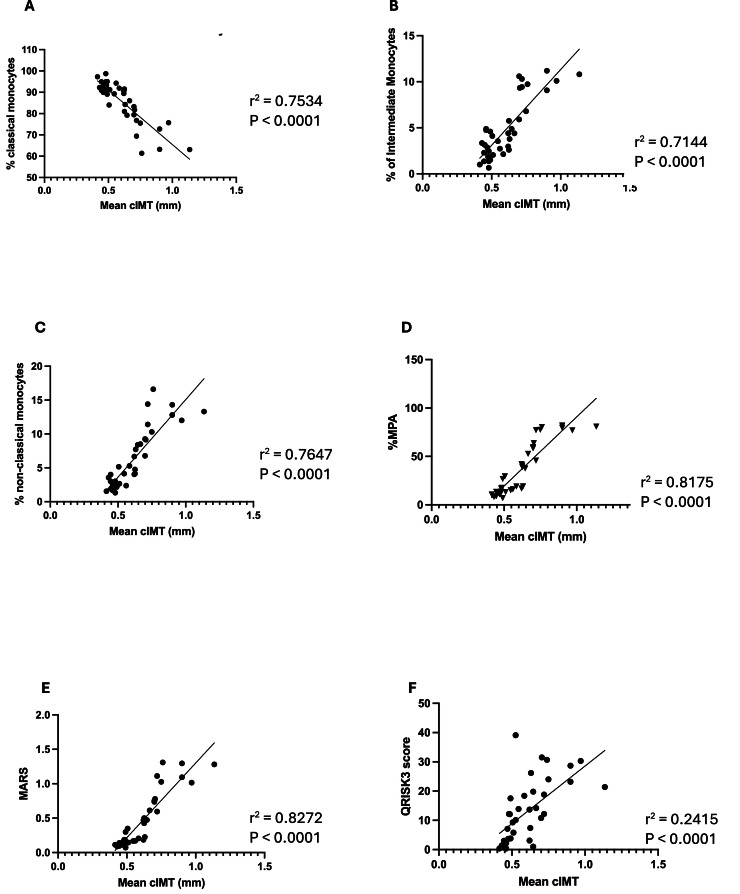

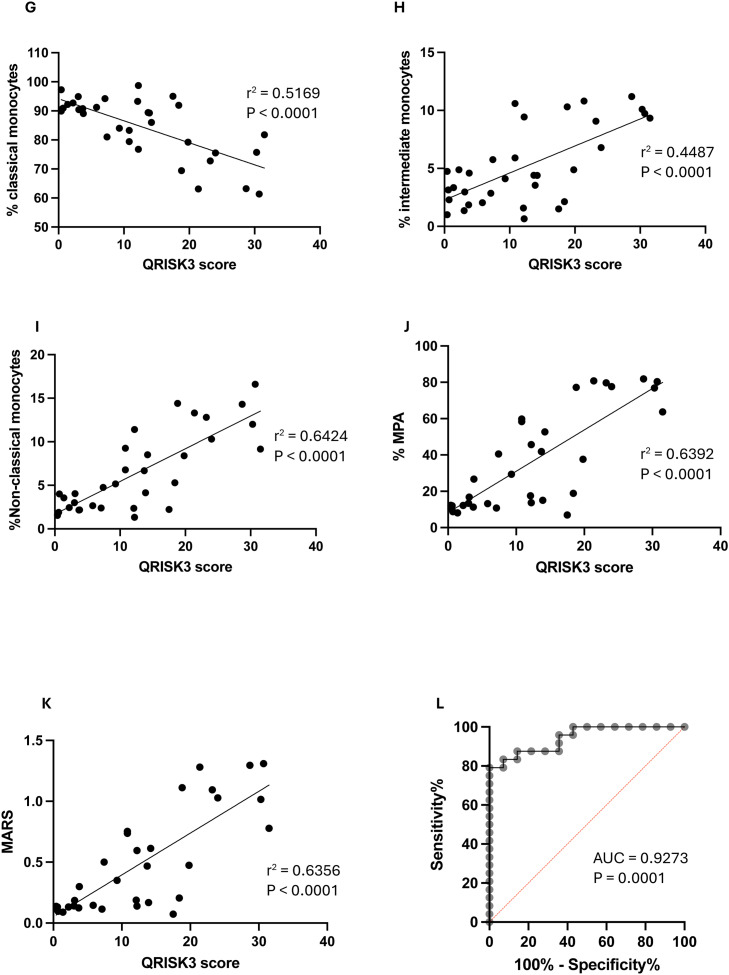

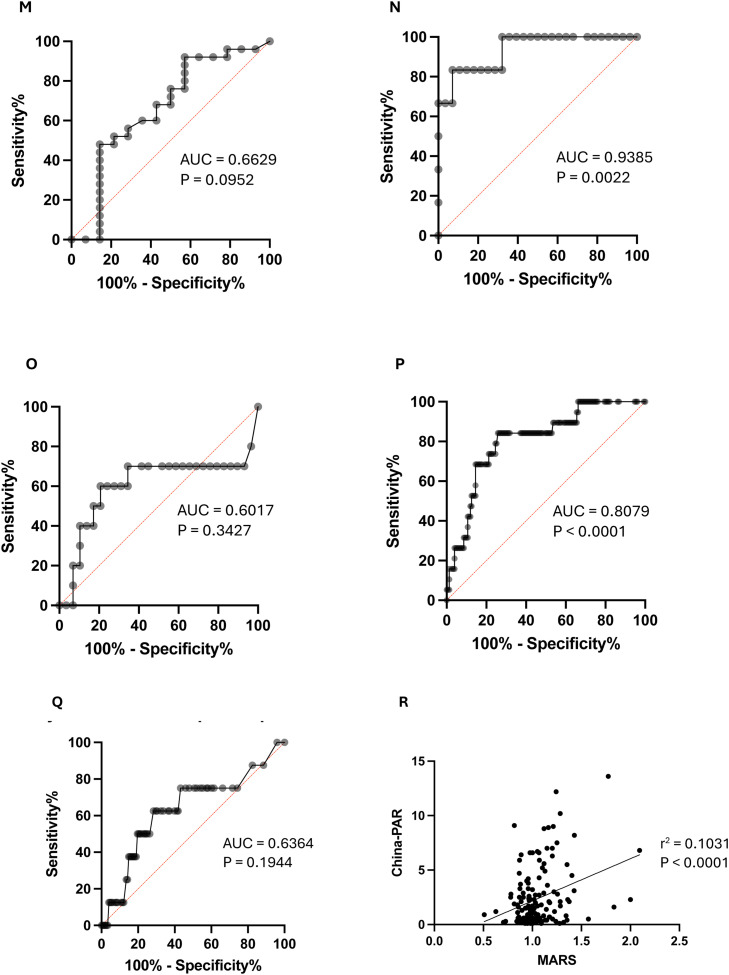
**A-F,** Linear regression analyses of the relationship between mean cIMT and classical (**A**), intermediate (**B**) and non-classical (**C**) monocytes, MPAs (**D**), MARS (**E**) and QRISK3 score (**F**) in the discovery cohort. **G-K,** Linear regression analyses of relationship between QRISK3 score and classical (**G**), intermediate (**H**) and non-classical (**I**) monocytes, MPAs (**J**) and MARS (**K**) in the discovery cohort. **l-O,** Receiver operating characteristic (ROC) curve analysis of predictive ability of MARS to predict high risk cIMT (**L**) or presence of plaque (**N**), and of QRISK3 score to predict high risk cIMT (**M**) or presence of plaque (**O**) in the discovery cohort. **P-Q,** ROC curve analysis of predictive ability of (**P**) MARS and (**Q**) China-PAR to predict the presence of plaque in the validation cohort. Also shown is the correlation analysis between MARS and China-PAR **(R)** in the validation cohort.

Of 39 participants, 10 had carotid plaque. Monocyte subsets showed distinct relationships with atherosclerosis (Fig. 1A-C): classical monocytes were inversely associated with cIMT, whereas intermediate and non-classical subsets showed positive associations. MPAs also correlated positively with cIMT (Fig. 1D). MARS demonstrated the strongest association with cIMT, the r^2^ considerably outperforming QRISK3 (Fig. 1E-F). Although QRISK3 correlated significantly with all monocytic subsets and with MPAs, the r² values were low (Fig. 1G-J), as was that for the correlation of QRISK3 with MARS (Fig. 1K).

MARS showed excellent discrimination of high-risk cIMT (Fig. 1L) and of carotid plaque (Fig. 1N), whilst QRISK3 showed no significant predictive ability high-risk cIMT (Fig. 1M) or carotid plaque (Fig. 1O). Combining QRISK3 with MARS did not improve performance.

### 3.2 Validation cohort

MARS was significantly higher in individuals with carotid plaque and reliably discriminated plaque presence (Fig. 1P), whilst China-PAR did not show significant predictive ability (Fig. 1Q). Correlation between MARS and China-PAR was weak (Fig. 1R).

## 4 Discussion

This study demonstrates that circulating monocyte phenotype and MPAs provide biologically informative markers of subclinical atherosclerosis and outperform conventional risk calculators. Traditional risk models estimate long-term risk based on clinical variables but do not incorporate measures of active vascular inflammation, limiting their utility at the individual level. In contrast, MARS reflects ongoing cellular processes central to atherogenesis, including monocyte activation and platelet–monocyte interactions.

The superior performance of MARS likely reflects integration of complementary biological signals, capturing both inflammatory activation and shifts in monocyte populations. This composite approach outperforms individual markers and suggests that multi-parameter indices provide more robust measures of disease biology.

Clinically, a blood-based biomarker such as MARS could help identify subclinical atherosclerosis through more targeted use of imaging, thereby improving individual risk stratification, and supporting more efficient clinical trial design by enriching populations with underlying disease. Monocytes play a key role in plaque initiation through endothelial adhesion and transmigration, while MPAs amplify inflammatory and thrombotic pathways [7]. The observed associations are therefore biologically plausible and extend prior findings in symptomatic populations to asymptomatic individuals.

Although imaging remains the reference standard for detecting atherosclerosis, it is not scalable for screening in most countries. A biomarker such as MARS could serve as a triage tool, identifying individuals most likely to benefit from imaging and preventive interventions.

Limitations of our study include the modest sample size of the discovery cohort and the cross-sectional design. However, findings were consistent across markers and replicated in an independent validation cohort. Furthermore, whereas risk calculators are used to predict the probability of cardiovascular events, they are not designed to predict the presence of subclinical atherosclerosis *per se* – although we would predict that the two should be strongly associated. Therefore, prospective large well powered studies are required to both replicate our findings and determine whether MARS truly predicts clinical events.

## 5 Conclusion

Monocyte phenotype and MPAs provide a mechanistically grounded biomarker of subclinical atherosclerosis. The composite MARS index identifies silent carotid disease with greater accuracy than established cardiovascular risk calculators across diverse populations. These findings support further evaluation of the potential utility of monocyte-based biomarkers for personalised cardiovascular risk assessment and targeted prevention.

## Sources of funding

FA was funded by a scholarship from Kuwait University, administered through the Kuwait Cultural Office (London, UK). This work was supported by a King’s British Heart Foundation Centre for Excellence Award [RE/18/2/34,213], and by The National Natural Science Foundation of China (grant number 82,200,299). The content is solely the responsibility of the authors and does not necessarily represent the official views of any funding agencies.

## Disclosures

The authors declare no conflicts of interest or relationships relevant to the contents of this paper to disclose.

## Author agreement

All authors have agreed to the submission of this manuscript. The manuscript has not been previously published nor is under consideration for publication elsewhere.

## CRediT authorship contribution statement

**Fatemah Almarri:** Writing – review & editing, Investigation, Formal analysis. **Shuaihua Qiao:** Writing – review & editing, Investigation, Formal analysis. **Jiale Gao:** Writing – review & editing, Investigation. **Li'na Kang:** Writing – review & editing, Investigation. **Soundrie Padayachee:** Writing – review & editing, Investigation. **Biao Xu:** Writing – review & editing, Supervision. **Ashish Patel:** Writing – review & editing, Writing – original draft, Supervision, Resources. **Albert Ferro:** Writing – review & editing, Writing – original draft, Supervision, Project administration, Funding acquisition, Formal analysis, Data curation, Conceptualization.

## Declaration of competing interest

The authors declare the following financial interests/personal relationships which may be considered as potential competing interests:

Albert Ferro reports financial support was provided by King’s College London. If there are other authors, they declare that they have no known competing financial interests or personal relationships that could have appeared to influence the work reported in this paper.
